# Effects of Wall Thickness Variation on Hydrogen Embrittlement Susceptibility of Additively Manufactured 316L Stainless Steel with Lattice Auxetic Structures

**DOI:** 10.3390/ma16062523

**Published:** 2023-03-22

**Authors:** Mahmoud Khedr, Atef Hamada, Walaa Abd-Elaziem, Matias Jaskari, Mahmoud Elsamanty, Jukka Kömi, Antti Järvenpää

**Affiliations:** 1 Mechanical Engineering Department, Faculty of Engineering at Shoubra, Benha University, Cairo 11629, Egypt; 2 Kerttu Saalasti Institute, Future Manufacturing Technologies (FMT), University of Oulu, 85500 Nivala, Finland; 3 Department of Mechanical Design and Production Engineering, Faculty of Engineering, Zagazig University, Zagazig 44519, Egypt; 4 Mechatronics and Robotics Department, School of Innovative Design Engineering, Egypt-Japan University of Science and Technology, Alexandria 21934, Egypt; 5 Materials and Mechanical Engineering, Centre for Advanced Steel Research, University of Oulu, 90014 Oulu, Finland

**Keywords:** hydrogen embrittlement, additive manufacturing, 316L stainless steel, auxetic structure, mechanical behavior, SEM

## Abstract

In the present study, the hydrogen embrittlement (HE) susceptibility of an additively manufactured (AM) 316L stainless steel (SS) was investigated. The materials were fabricated in the form of a lattice auxetic structure with three different strut thicknesses, 0.6, 1, and 1.4 mm, by the laser powder bed fusion technique at a volumetric energy of 70 J·mm^−3^. The effect of H charging on the strength and ductility of the lattice structures was evaluated by conducting tensile testing of the H-charged specimens at a slow strain rate of 4 × 10^−5^ s^−1^. Hydrogen was introduced to the specimens via electrochemical charging in an NaOH aqueous solution for 24 h at 80 °C before the tensile testing. The microstructure evolution of the H-charged materials was studied using the electron backscattered diffraction (EBSD) technique. The study revealed that the auxetic structures of the AM 316L-SS exhibited a slight reduction in mechanical properties after H charging. The tensile strength was slightly decreased regardless of the thickness. However, the ductility was significantly reduced with increasing thickness. For instance, the strength and uniform elongation of the auxetic structure of the 0.6 mm thick strut were 340 MPa and 17.4% before H charging, and 320 MPa and 16.7% after H charging, respectively. The corresponding values of the counterpart’s 1.4 mm thick strut were 550 MPa and 29% before H charging, and 523 MPa and 23.9% after H charging, respectively. The fractography of the fracture surfaces showed the impact of H charging, as cleavage fracture was a striking feature in H-charged materials. Furthermore, the mechanical twins were enhanced during tensile straining of the H-charged high-thickness material.

## 1. Introduction

The recent trend towards clean energy is the main priority for the world due to the energy crisis. Shifting to a carbon-neutral economy in mitigating climate change increases worldwide interest in clean energy sources. Hydrogen (H) is considered an optimum green alternative to traditional fossil fuel sources in terms of reducing carbon footprint to enhance environmental sustainability [1,2]. H can be produced by decoupling H atoms from their carriers, such as water [3,4]. However, the storage of H and/or being present in an H environment negatively affects the strength and ductility of different steel grades [5,6,7,8,9,10] because the H accelerates crack growth rate and so shortens service life; this phenomenon is known as hydrogen embrittlement (HE) susceptibility. Therefore, addressing the challenges associated with HE is crucial for realizing the full potential of H as a clean energy source.

Additive manufacturing (AM) is a promising production method that can fabricate complex-shaped parts by building layers of a specific material in three dimensions (3D) [11,12]. Among various AM techniques, laser powder bed fusion (LPBF) is a widely used technique for the 3D printing of metals [13,14]. Nevertheless, Baek et al. [15] reported that the HE resistance of the metals differs depending on the processing method, for example, whether subtractive or additive techniques are used. Therefore, the HE susceptibility of metals printed by the LPBF requires more research to evaluate their sensitivity towards working in an H-surrounded environment. Addressing this research gap will contribute to developing robust and reliable metals, fabricated by AM processes, that are suitable for producing parts that are resilient to HE and can function efficiently in H-rich environments. Ultimately, this could help to accelerate the adoption of clean energy sources and contribute to global efforts to mitigate climate change.

316L stainless steel (SS) has found widespread use in various industries due to its exceptional strength, ductility, and resistance to corrosion [16,17]. Traditional manufacturing methods used to produce 316L SS components may limit the mechanical properties. However, the advent of AM techniques, such as LPBF, has allowed the fabrication of 316L-SS components with improved mechanical properties. The cellular structures formed during the LPBF process promote mechanical twinning and contribute to the increased strength and ductility of 316L-SS components printed by LPBF compared to those produced using traditional manufacturing methods [18]. However, 316L-SS suffers from HE when subjected to an H medium.

Various studies have been conducted on the HE susceptibility of 3D-printed 316L-SS [19,20,21,22,23,24]. These studies have found that volumetric defects, sub-grains in the cellular structure, and the melt pool boundaries present in selective laser melted (SLMed) 316L-SS act as potential trapping sites for H atoms. Moreover, the stain fields around the edge dislocations work as reversible H-trapping sites (with activation energy less than 60 kJ/mol), which promotes the HE susceptibility [21,25]. Nevertheless, the HE resistance of 316L-SS produced by the LPBF technique is better than its counterparts produced by conventional methods due to the enhanced strength originating from the existing sub-grains in the cellular structures as well as the austenite being more stable in the LPBF specimens [21,22]. The latter property minimizes the strain-induced transformation of austenite into martensite in the LPBF structures compared with the conventional 316L-SS [15,21,26], since H reduces the stacking fault energy of the austenitic steels [27,28].

The manipulation of the design parameters at the lattice form can lead to extraordinary mechanical behavior of the engineering parts. For instance, materials with auxetic structures show positive extension in axial and lateral directions when subjected to tensile loading and vice versa when compressed [29,30]. This property is utilized in various engineering applications, such as acoustic isolators, shock absorbers, etc. [31,32]. Metals with auxetic structures are produced by nonconventional manufacturing processes such as LPBF [33,34,35]. The structures with lattice form are a type of cellular structure that are formed by repeating a unit cell in 3D; their mechanical behavior can be optimized by altering the unit cell dimensions [36,37]. A recent review article presented comprehensive literature on the energy consumption and quality characteristics of AM processes [11]. One of the most interesting findings in the literature is the scarcity of studies concerning the additive manufacturing of thin-walled structures. The effect of the wall thickness variation on the HE susceptibility of different steel grades has been reported [38,39,40]. Rhode et al. [38] concluded that, as the wall thickness of the H-charged specimens increased, the H-spread through the specimens was delayed, resulting in an increased diffused hydrogen breakthrough time compared with the thinner samples, which was ascribed to the increased length of the H diffusion path. However, the number of hydrogen trapping sites increases in the thicker specimens. Similarly, Li et al. [39] confirmed that thicker-walled specimens are more susceptible to HE than thinner ones. In contrast, it was concluded that the strength of 3D-printed 316L SS is negatively affected by thickness reduction [41].

316L-SS with lattice/cellular structure has shown promising potential in energy absorption and lightweight manufacturing applications, such as in the automobile and aerospace industries, due to its ability to reduce fuel consumption rates [42]. However, there is a lack of knowledge about the mechanical behavior of lattice structures when exposed to H environments, although the surface area of the lattice/cellular structures subjected to H atoms is greater than that of their solid form counterparts. Furthermore, the HE susceptibility of auxetic lattice structures in the 3D form has not been previously investigated. This is particularly relevant with regard to increasing interest in using H as a clean fuel source. Therefore, the present study aims to investigate the HE susceptibility of 316L-SS printed with an auxetic lattice structure using the LPBF technique. The studied AM 316L-SS auxetic structures were printed with various strut thicknesses, specifically 0.6, 1, and 1.4 mm.

## 2. Experimental Procedures

### 2.1. Materials

The lattice structures, with various strut thicknesses, made of 316L-SS were printed by the LPBF technique using a selective laser melting (SLM) machine (Model: SLM 280HL LPBF, SLM Solutions, Lübeck, Germany). Volumetric energy was kept constant, at 70 J·m^−3^, during the printing of the different strut thicknesses; the hatching lines were printed at a laser power and a scanning speed of 200 W and 0.8 mm/s, respectively. The powder was provided by the Lübeck company, Germany. Table 1 shows the chemical composition of the printed 316L-SS.

**Figure 1:**
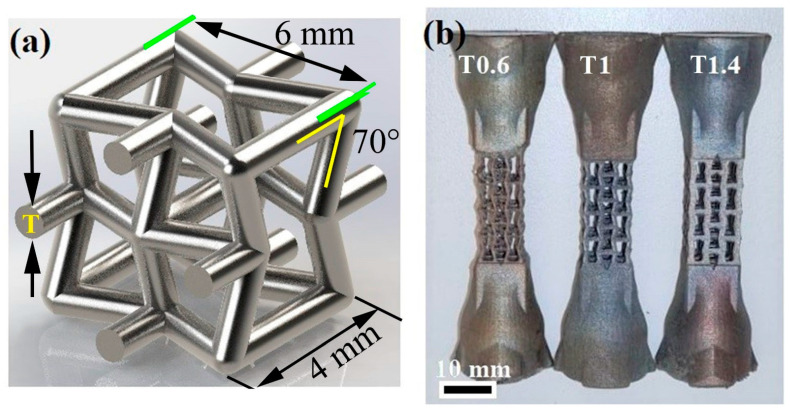
(**a**) Shape and dimensions of one unit cell of the printed 316L lattice structure; (**b**) macrograph of H charging sample geometry showing the lattice structure with different thicknesses. T refers to the strut thickness.

The form of the printed 316L-SS samples is shown in Figure 1. The shape and dimensions of one unit cell of the printed lattice structure are displayed in Figure 1a, which shows a re-entrant pattern with a 6 mm side and a 4 mm base. The angle between the side and the base (re-entrant orientation angle) is 70°. Various patterns with different thicknesses (t = 0.4, 0.6, and 1.6 mm) were printed. Figure 1b shows the geometry of the H-charging testing samples, which underwent subsequent tensile testing. Solid heads were printed with a lattice structure to fit into the grips of the tensile testing machine. The cross-section of the lattice structure was 25 × 12 × 12 mm^3^.

The tensile tests were carried out after H charging, within a delay time of 5 min, by a Zwick/Roell (Z100, ZwickRoell GmbH, Ulm, Germany) universal testing machine at a slow strain rate of 4 × 10^−5^ s^−1^. The fracture surfaces of the non-charged and H-charged 316L-SS were examined by secondary electron imaging with a scanning electron microscope (SEM; Zeiss-UltraPlus, Jena, Germany) at 15 KV.

### 2.2. H-Charging Setup

**Figure 2:**
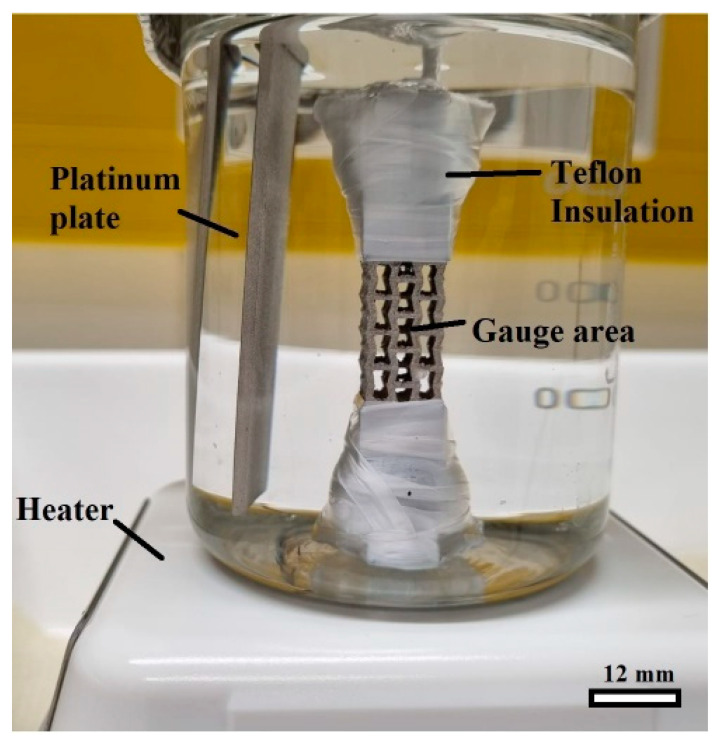
A photograph showing the setup during H charging of the auxetic structure.

The electrochemical H-charging process was conducted using a solution of 3% NaOH and 3 g/L CH_4_N_2_S (Thiourea) for 24 h at 80 °C under a constant current density of 20 mA·cm^−2^. The temperature was monitored by a mercury thermometer during the H-charging process. A platinum plate was used as a counter electrode. During the H charging, the gauge area was in direct contact with the solution, while the other surfaces were insulated by Teflon. A macrograph of the setup used during the H-charging process in the present study is shown in Figure 2.

H is generated in the current study by the electrochemical reactions on the surface of the 316L-SS specimens as H gas has the ability to be adsorbed and dissociated on the steel surface to produce atomic H. Subsequently, the H atoms diffuse into the steel through the structure defects.

### 2.3. Microstructure Characterization

The surface topography of the 3D printed 316L-SS material with lattice structure was examined by laser scanning confocal microscopy (Model: Keyence/VK-X200, Keyence, Osaka, Japan). The electron backscatter diffraction (EBSD) technique, using an FEG-SEM (model: Carl Zeiss Ultra plus: Oberkochen, Germany), was employed to study the effect of H charging on the microstructure and deformation mechanism during tensile straining. EBSD examination, after the tensile testing, of the H-charged specimens was conducted at an accelerating voltage of 15 kV, a step size of 0.5 μm, and a working distance of 12–15 mm.

## 3. Results

### 3.1. Surface Morphology

**Figure 3:**
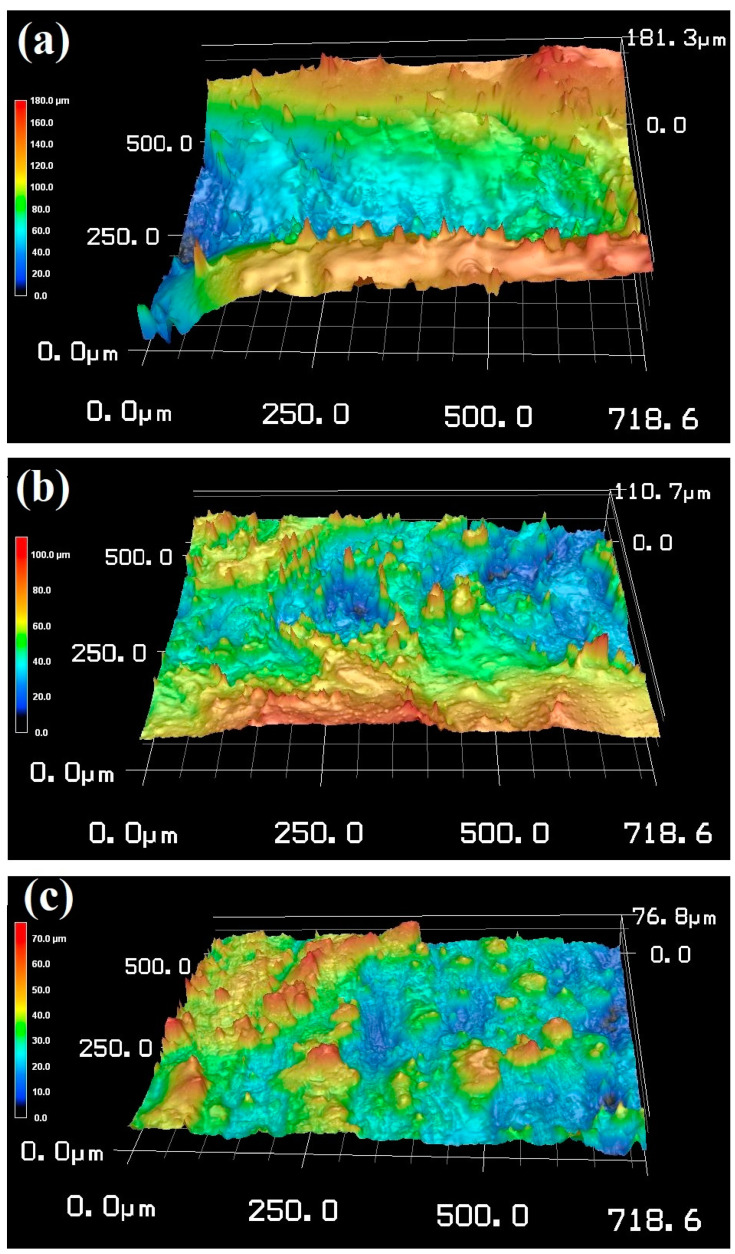
Surface topography and the corresponding surface roughness profile along the yellow lines measured by laser confocal scanning microscope. (**a**) T0.6, (**b**) T1, and (**c**) T1.4.

The laser microscope 3D profiles of the surface topography of the 3D-printed 316L lattice structures at various thicknesses (T0.6, T1, and T1.4) are displayed in Figure 3. The roughness measurements were taken along several lines over the surface. The topography of the surface increased as the strut thickness was reduced. For instance, the average surface roughness values (Ra) of T0.6, T1, and T1.4 were 21 ± 4, 10 ± 2, and 8 ± 2 μm, respectively. In agreement with Ahuja et al. [40] and Niendorf et al. [43], the surface roughness of the AM steel increases with decreasing thickness. It is worth noting that surface roughness affects the H penetration of the metals, as reported in the literature [44,45].

### 3.2. Mechanical Tensile Properties: Effect of H-Charging

**Figure 4:**
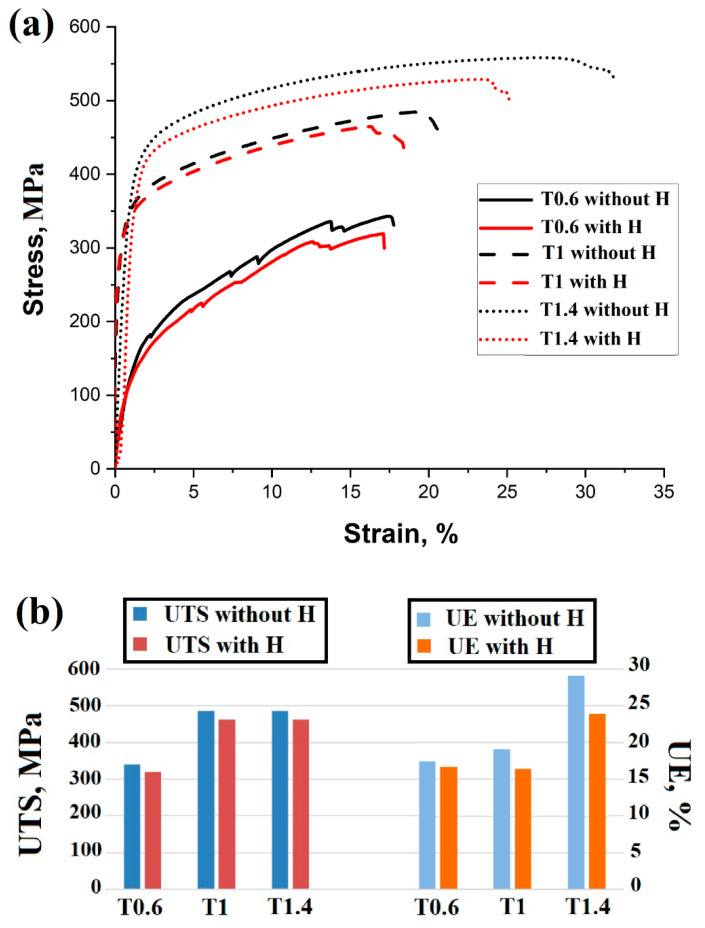
(**a**) Engineering stress–strain curves for AM 316L-SS with lattice structure and different thicknesses: with and without H charging; (**b**) graphical representation of UTS and UE% before and after H charging.

Figure 4a displays the engineering stress–strain curves of T0.6, T1, and T1.4 before and after electrochemical charging with H. Figure 4b shows graphical representations of the ultimate tensile strength (UTS) and uniform elongation (UE) % of the auxetic structures with the different thicknesses before and after H charging. It is evident that the mechanical properties of the auxetic structure without H charging are significantly affected by thickness, as strength and ductility increase with increasing thickness of the printed auxetic structures. Table 2 illustrates the mechanical properties, such as yield strength (YS), UTS, and UE, of the auxetic structures. It can be seen that, as thickness increased from 0.6 to 1.4 mm, the UTS increased from 340 ± 10 to 550 ± 11 MPa. In other words, the strength of the auxetic structure increased by ~62% when the thickness was doubled. Meanwhile, the UE increased by ~66%

With H charging, the strength of the studied thicknesses was slightly decreased, as shown by the flow curves in Figure 4. For example, for T1.4 the UTS has decreased from 550 to 523 MPa, roughly a 5% reduction. This confirms the high resistance of 316L-SS to HE susceptibility. However, the reduction in UE increases with thickness. For instance, T1.4 displayed the highest percentage of UE reduction, approximately 17.6%.

Unlike the H-free auxetic specimens, the reduction in the strength of the H-charged specimens is not dependent on the thickness of the struts. However, the reduction in the UE % is greatly affected by the strut thickness. It is clear that the sensitivity of the auxetic structures to HE susceptibility is increased with increasing thickness.

### 3.3. Fractography

**Figure 5:**
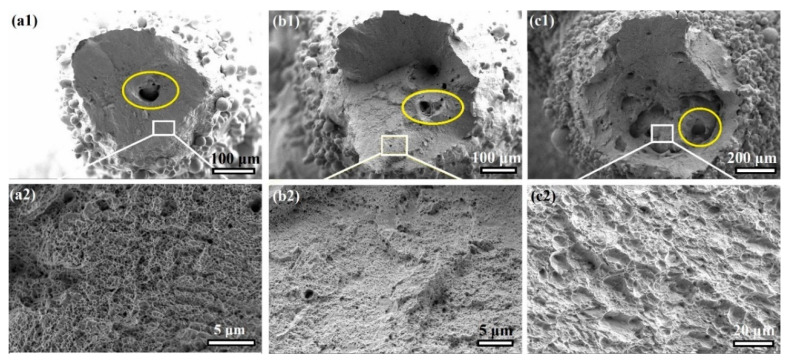
The fracture surfaces of the H-free specimens. SEM images of (**a**) T0.6, (**b**) T1, and (**c**) T1.4 after tensile testing. The magnification views in (**a2**,**b2**,**c2**) are selected as indicated by the white rectangles in (**a1**,**b1**,**c1**).

Figure 5 displays the fractography of the H-free specimens after tensile testing. Cup-and-cone-type fracture dominated the fracture surfaces of the various thicknesses. This indicates ductile fracture of the auxetic structures regardless of the thickness, as shown in Figure 5a1–c1. The magnified views (inside the white squares) of the fracture surfaces display the dimple feature, which confirms the ductile fracture type, as shown in Figure 5a2–c2. Ductile fracture of AM 316L-SS has been reported by several previous studies, which stated that dimples are promoted due to microvoid coalescence after fracture of the solid 316L-SS [46,47]. A striking feature is the presence of pores with different sizes in the auxetic structures, highlighted by the yellow ovals. The pores range in size between 30 and 60 μm. It is reported that the appearance of the pores in AM products is related to different uncontrolled printing conditions, such as gas absorption when excessive laser energy is applied [48], incomplete fusion of the powder due to insufficient energy input [49], laser beam penetration of a solid established layer, or inadequate penetration of molten metal through a previously formed layer during printing [50].

**Figure 6:**
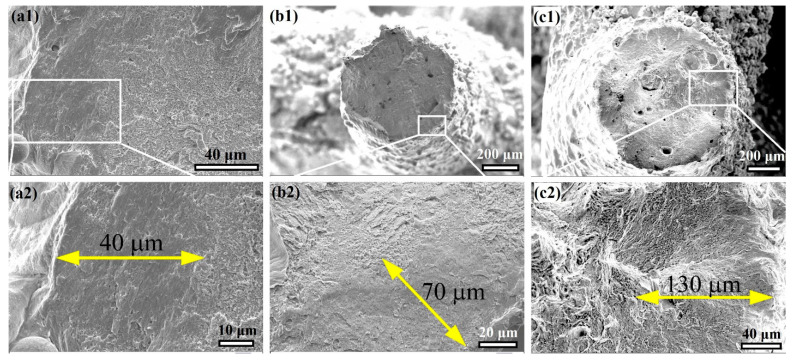
The fracture surfaces of the H-charged specimens. SEM images of (**a**) T0.6, (**b**) T1, and (**c**) T1.4 after tensile testing. The magnification views in (**a2**,**b2**,**c2**) are selected as indicated by the white rectangles in (**a1**,**b1**,**c1**). The yellow arrows refer to the depth of the cleavage facets beneath the edges of the specimens.

Figure 6 shows the fracture surface topography of the H-charged specimens after tensile testing. The fracture surface characteristics were changed from dimpled ductile features in the H-free specimens to quasicleavage-type fractures near the edge. However, the central region has dimples mixed with transgranular features. Hong et al. [19] showed a similar topography of H-charged AM 316L.

It is evident that the size of cleavage zone has changed with the auxetic strut thickness. For instance, the H-charged T0.4, T1, and T1.4 specimens displayed cleavage zone sizes of 40 ± 10, 70 ± 10, and 130 ± 15 μm, respectively, as shown in Figure 6a2–c2. It seems that, as the thickness of the lattice structure increases, HE susceptibility is increased in the AM 316L-SS.

## 4. Discussion

### 4.1. Effect of Thickness Change on HE Susceptibility

**Figure 7:**
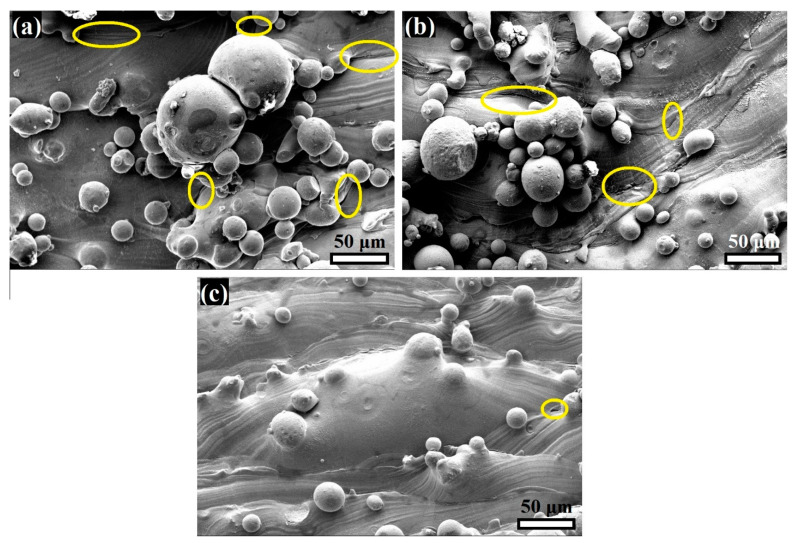
SEM images of the surface morphology of the printed 316L-SS specimens: (**a**) T0.6, (**b**) T1, and (**c**) T1.4. The yellow ovals refer to the surface flaws.

Figure 7 shows the surface morphology of the printed T0.6, T1, and T1.4 (before H charging). The balling effect, due to partially melted powder, is clearly evident on the surface of the struts. The size of the balling is increased with a reduction in strut thickness. For instance, the sizes of the surface balls are 85, 50, and 35 μm in T0.6, T1, and T1.4, respectively. Therefore, the surface roughness increased as strut thickness was reduced (see Figure 3). Furthermore, more surface flaws occur as the thickness is reduced, highlighted by the yellow ovals in Figure 7. This is attributed to the superior cooling during printing of the thinner strut structure [26,51], which promotes high levels of the lattice strain due to the great variation in edge shrinkage, resulting in the formation of more microcracks in T0.6 than T1.4.

There are two competing factors which affect the HE susceptibility of AM316L SS: the surface roughness and the hydrogen trapping sites [19,20,21,22,23,45]. Hydrogen atoms effectively diffuse into the rough surface as the surface flaws and other imperfections, such as pores, serve as entrances for the H atoms, as reported in the literature [24,44,45,52]. H-trapping sites are also significant; these are reversible and irreversible traps in the structure, which capture H atoms inside the material. The thin sections have more surface flaws than the thick sections, which act as irreversible H-trapping sites. Therefore, the surface flaws cause irreversible H uptake, and a large amount of energy (more than 60 kJ/mol) is then required for the H to be diffused out [21,25]. Consequently, HE susceptibility decreases with an increase in the surface flaws on the thin sections, such as in the auxetic structure of T0.6.

Whereas the thickest strut, T1.4, displayed fewer surface flaws, the mechanical properties were more affected by H-charging. This can be attributed to reversible trapping sites, such as dislocation cores [23], which are increased with increasing cross-section, as reported in the literature [38,39]. This is in an agreement with the mechanical properties illustrated in Table 2. We could suggest that the current study, qualitatively, showed the effect of H charging on the auxetic structures with various thicknesses. However, thermal desorption spectroscopy (TDS) is needed to measure the H-trapping content. This would be an interesting area of future study.

Figure 5 shows that the yield strength (YS) was negatively affected by H charging. It is well known that a reduction in the YS of austenitic stainless steels is related to enhanced dislocation movements via the dislocation climb mechanism [53]. The presence of H atoms promotes the dislocation climb, i.e., activation of the HELP mechanism, by reducing the interaction energy between glissile dislocations and their obstacles [6,9,28,54] in the early stages of the plastic deformation of austenitic stainless steel, regardless of the thickness change. Thus, the YS of the AM 316L-SS was reduced after H charging.

As shown in Table 2, the UTS of the H-charged lattice structures were slightly decreased, 5 and 6% reductions were recorded. However, the reduction in the UE % of the H-charged specimens is considerable and directly proportional to the thickness. This agrees with the literature, in which the HE of SLMed 316L-SS [19,24] and 304-SS [26] were investigated, the UTS was minimally affected, whereas elongation reduction has been intensively reported after H charging.

The existence of the reversible H atoms, locally, raises the stress concentration at the structure interfaces, resulting in the promotion of crack initiation, and sudden fracture occurs after reaching the UTS value, known as the hydrogen-induced cracking (HIC) effect [21,26,55]. Moreover, the deterioration in ductility is directly proportional to the thickness increase, which is attributed to the increase in reversible H-trapping sites as the thickness increases. It has been reported in the literature [38,39] that reversible H-trapping sites are increased with an increase in thickness, and, consequently, more diffusible H exists in the T1.4 specimen. In addition, the depth beneath the edges affected by the existence of H was increased with increasing thickness, which was confirmed by fracture surface fractography, as shown in Figure 6a2–c2. This is apparent from the cleave zone size in the fracture surface (see Figure 6). Clearly, irreversible H traps are increased in the thin struts due to the greater promotion of surface flaws than occurs on the thick strut, which eliminates HE susceptibility in the T0.6 specimen. In other words, the greater the strut thickness, the higher the reversible H trapping, which increases cleavage zone size and decreases the ductility of the AM 316L-SS with lattice structure.

### 4.2. Microstructure Evolution in the H-Charged Specimens

**Figure 8:**
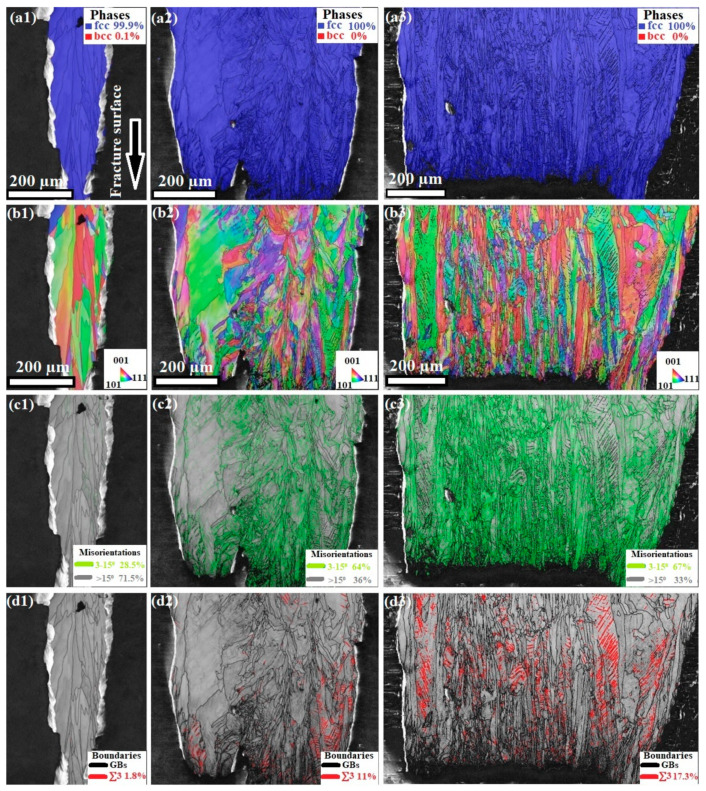
EBSD maps of H-charged microstructures of auxetic 316L-SS with various thicknesses: T0.6 (**1**), T1 (**2**), and T1.4 (**3**). (**a**) Phase maps showing (γ-FCC) in blue; (**b**) inverse pole figure (IPF) maps; (**c**) misorientation boundary maps showing LAGBs in green, and (**d**) boundary maps showing Σ3 twin boundaries in red.

Figure 8 shows the EBSD maps of the microstructures of the auxetic 316L-SS specimens that have undergone H charging and subsequent tensile testing. The microstructure examination was conducted at the gauge area near the fracture surface. It is evident that the phase maps, shown in Figure 8a1–a3, of the H-charged microstructures display only blue color, which confirms a uniaustenitic phase in the structure, i.e., fully austenite γ-FCC without phase transformation, regardless of the strut thickness variation. This is attributed to the high stability of the 316L-SS against phase transformation that is related to the stacking fault energy (SFE). It has been reported that the SFE of 316L-SS is 27 mJ·m^−2^, as stated by Jaskari et al. [56]. It has been established that the SFE controls the deformation mechanism of austenitic stainless steel. Martensitic transformation is favorable at a SFE values less than 18 mJ·m^−2^. However, at an intermediate SFE value, 18–45 mJ·m^−2^, a mechanical twinning deformation mechanism is favorable [53,57,58].

Figure 8b shows the inverse pole figure (IPF) maps obtained by EBSD. The T0.6 specimen displayed more homogeneous orientations than T1 and T1.4, as the T0.6 displayed preferred orientation of the grains along [001] and [101], whereas T1 and T1.4 showed random grain orientation.

Figure 8c shows the misorientation boundary maps obtained by EBSD. It can be seen that the low angle grain boundaries (LAGBs), which are colored green and range from 3° to 15°, increase with increasing thickness. The volume fraction of the LAGBs in T0.6, T1, and T1.4 are 28.5, 64, and 67%, respectively. The increase in the volume fraction of the LAGBs is a result of enhanced localized plasticity during the tensile straining, due to dislocation slip [59] caused by H atoms. Thus, LAGBs are induced inside the grain structure to accommodate the plasticity, which agrees with the literature [24]. This confirms that the absorption of the reversible H atoms in the thick struts is greater than in the thinner struts.

Figure 8d shows the boundary maps (BM) via EBSD; the grain boundaries are colored black, and the ∑3 boundaries (with 60° rotation) are defined in red. The volume fraction of the ∑3 boundaries measured 1.8%, 11%, and 17.3% in the H-charged microstructures of T0.6, T1, and T1.4, respectively. Moreover, by comparing the BM with the IPF, it is clear that twinning was promoted in [001] and [101] oriented grains, which is in agreement with the findings reported by Gao et al. [60].

It is well known that the promotion of mechanical twins during deformation enhances the plasticity of a material. The T1.4 auxetic structure showed the highest fraction of ∑3. Hence, this strut thickness displayed more plasticity, as shown in the flow curves (see Figure 4).

The formation of ∑3 boundaries, i.e., twin boundaries, is related to the SFE value, since mechanical twinning is favorable in the range of 18 to 45 mJ·m^−2^ [53,57,58]. Yamada et al. [27] reported that H charging reduces the SFE, which enhances mechanical twinning in austenitic steels. Consequently, the auxetic structure with the highest strut thickness, T1.4, displayed a significant fraction of ∑3 boundaries owing to reduced SFE.

Whereas the mechanical twins enhance the strength–ductility combination in 316L-SS, H enhances cracking along twin boundaries [61]. H induces a localized stress concentration at the twin–twin boundary intersections. These sites are crack initiation sources and expedite fracture [62,63]. Therefore, with an increase in the fraction of mechanical twins in the thick strut, T1.4, the number of crack initiation sites increases. Consequently, more microcracks are induced, which decreases the ductility. This can be seen in the flow curves in Figure 4, which show that T1.4 has the highest reduction in UE %.

## 5. Conclusions

In the present study, auxetic lattice 316L-SS with different thicknesses (T0.6, T1, T1.4) was additively manufactured by the LPBF technique. Subsequently, the 3D printed structures of 316L-SS underwent an electrochemical H-charging process at 80 °C to investigate its impact on mechanical properties and microstructures. The following conclusions were drawn:

(1) The surface morphology of the auxetic lattice structure was affected by the thickness variation. The minimum strut thickness (T0.6) displayed the highest surface roughness, Ra 21 μm, with significant surface flaws.
(2) The mechanical strength of the auxetic structures slightly decreased, about 5–6%, for all strut thicknesses. However, the ductility (UE) of the highest strut thickness (T1.4) showed a considerable reduction, about 17.6%, with H charging.
(3) The fracture surfaces of the noncharged 316L-SS are better than that of their counterparts in the H-charged sample. Whereas the fracture surfaces of the noncharged material mainly showed a dimple feature, the H-charged material exhibited a cleavage fracture region, which is due to the impact of HE susceptibility.
(4) No phase transformation in the H-charged material occurred; however, the mechanical twinning deformation mechanism was enhanced in the high strut thickness materials. The fractions of Σ3 boundaries were 1.8% and 17.3% in T0.6 and T1.4, respectively.

## Figures and Tables

**Table 1:** The chemical composition of the printed 316L stainless steel.

Element	C	Cr	Ni	Mn	Mo	Si	Ti	Nb	Fe
Wt.%	0.02	17.7	12.9	0.6	2.5	0.7	0.01	0.02	Bal.

**Table 2:** Mechanical properties of AM 316L-SS with different thicknesses before and after H charging.

Specimen	Without H			With H			UTS Reduction, %	UE Reduction, %
	YS, MPa	UTS, MPa	UE, %	YS, MPa	UTS, MPa	UE, %		
T0.6	132 ± 3	340 ± 6	17.4 ± 0.1	125± 4	320 ± 7	16.7 ± 0.1	6	4
T1	257 ± 6	486 ± 10	19.1 ± 0.4	245 ± 5	462 ± 10	16.4 ± 0.5	5	14
T1.4	299 ± 7	550 ± 10	29 ± 0.3	284 ± 5	523 ± 10	23.9 ± 0.3	5	17.6

## Data Availability

Data will be made available on request.
